# Analysis of sex-based differences in clinical and molecular responses to ischemia reperfusion after lung transplantation

**DOI:** 10.1186/s12931-021-01900-y

**Published:** 2021-12-22

**Authors:** Lourdes Chacon-Alberty, Shengbin Ye, Daoud Daoud, William C. Frankel, Hassan Virk, Jonathan Mase, Camila Hochman-Mendez, Meng Li, Luiz C. Sampaio, Doris A. Taylor, Gabriel Loor

**Affiliations:** 1 Department of Regenerative Medicine, Texas Heart Institute, Houston, TX USA; 2 Department of Biostatistics, Rice University, Houston, TX USA; 3 Division of Cardiothoracic Transplantation and Circulatory Support, Michael E. DeBakey Department of Surgery, Baylor College of Medicine, Houston, TX USA; 4 Department of Cardiopulmonary Transplantation and Center for Cardiac Support, Texas Heart Institute, 6770 Bertner Ave, Suite 355-K, Houston, TX 77030 USA; 5 Present Address: Division of Infectious Diseases, Department of Internal Medicine, Center for Antimicrobial Resistance and Microbial Genomics (CARMiG), University of Texas Health Science Center at Houston, Houston, TX USA; 6 Present Address: Department of Advanced Cardiopulmonary Therapies and Transplantation, University of Texas Health Science Center at Houston, Houston, TX USA; 7 Present Address: RegenMedix Consulting, Houston, TX USA

**Keywords:** Lung transplantation, Sex differences, Gender differences, Primary graft dysfunction, Cytokines, Inflammation

## Abstract

**Background:**

Sex and hormones influence immune responses to ischemia reperfusion (IR) and could, therefore, cause sex-related differences in lung transplantation (LTx) outcomes. We compared men’s and women’s clinical and molecular responses to post-LTx IR.

**Methods:**

In 203 LTx patients, we used the 2016 International Society for Heart and Lung Transplantation guidelines to score primary graft dysfunction (PGD). In a subgroup of 40 patients with blood samples collected before LTx (T0) and 6, 24, 48 (T48), and 72 h (T72) after lung reperfusion, molecular response to IR was examined through serial analysis of circulating cytokine expression.

**Results:**

After adjustment, women had less grade 3 PGD than men at T48, but not at T72. PGD grade decreased from T0 to T72 more often in women than men. The evolution of PGD (the difference in mean PGD between T72 and T0) was greater in men. However, the evolution of IL-2, IL-7, IL-17a, and basic fibroblast growth factor levels was more often sustained throughout the 72 h in women. In the full cohort, we noted no sex differences in secondary clinical outcomes, but women had significantly lower peak lactate levels than men across the 72 h.

**Conclusions:**

Men and women differ in the evolution of PGD and cytokine secretion after LTx: Women have a more sustained proinflammatory response than men despite a greater reduction in PGD over time. This interaction between cytokine and PGD responses warrants investigation. Additionally, there may be important sex-related differences that could be used to tailor treatment during or after transplantation.

**Supplementary Information:**

The online version contains supplementary material available at 10.1186/s12931-021-01900-y.

## Background

Studies of clinical outcomes after lung transplantation have shown a survival benefit for female versus male recipients, for reasons that remain unclear [1–3]. One potential explanation relates to sex-based differences in the pathogenesis of primary graft dysfunction (PGD). PGD is a leading cause of early morbidity and mortality after lung transplantation and portends poor late outcomes [4, 5]. The pathogenesis of PGD involves severe intraoperative ischemia–reperfusion (IR) injury resulting in alveolar inflammation and diffuse alveolar damage [6]. The molecular mechanisms underlying this process are complex, involving a biphasic response to which multiple immune cell lines contribute [7, 8].

Sex chromosome genes and sex hormones play a central role in immune regulation [9, 10]. The X chromosome contains the greatest density of immune-related genes in the whole genome [11]. In terms of sex hormones, testosterone (a suppressant) and estrogen (a stimulant) are thought to exert diametric influences on the immune response [12]. Whether sexual dimorphism in the immune and inflammatory response contributes to the pathogenesis of PGD and differential clinical outcomes after lung transplantation has not previously been explored. The objective of the current study was to compare clinical and molecular responses to IR after lung transplant in men versus women at a single center.

## Methods

### Study population

This study (Protocol Number H-42256) was approved by the Institutional Review Board for Human Subject Research for Baylor College of Medicine (BCM IRB). The BCM IRB is organized, operates, and is registered with the United States Office for Human Research Protections according to the regulations codified in the United States Coded of Federal Regulations at 45 CFR 46 and 21 CFR 56. The BCM IRB operates under the BCM Federal Wide Assurance No. 00000286, as those of hospital and institutions affiliated with the College. For prospective blood collection, all patients were consented. For the retrospective portion of the cohort, waiver of consent was approved. The data were anonymized for the privacy of the participants.

**Fig. 1:**
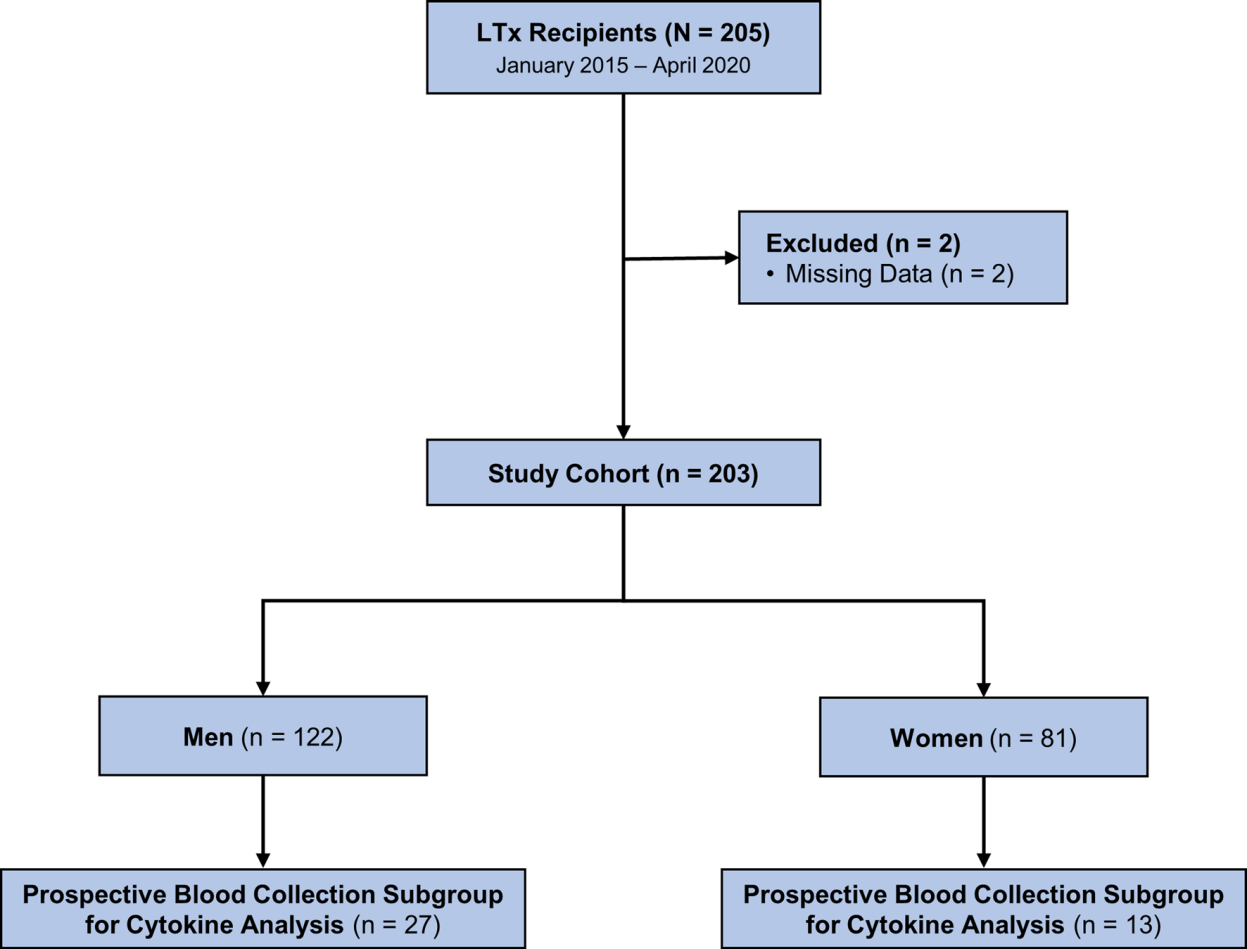
CONSORT diagram for the study cohort

Between January 2015 and April 2020, 205 consecutive patients underwent single or bilateral lung transplantation at our institution. Two patients were excluded because of missing data, leaving 203 total patients in the full cohort. Within this cohort, a subgroup of 40 consented patients had peripheral blood samples collected for cytokine analysis (Fig. 1). We use extracorporeal life support (ECLS) for ventilatory or hemodynamic support in patients who do not tolerate single-lung ventilation or a test clamping of the pulmonary artery intraoperatively. We use ECLS prophylactically in patients with severe pulmonary artery hypertension. Our preferred method of ECLS has gradually evolved from cardiopulmonary bypass to extracorporeal membrane oxygenation (ECMO). Our standard immunosuppression protocol was used for all patients in the study and included induction therapy with steroids, mycophenolate, and tacrolimus. All DCD donors included in our study were in Maastricht category 3.

### Study outcomes

Our primary aim was to compare clinical and molecular responses to IR after lung transplant in men versus women. The clinical response was described by using the PGD score according to the 2016 International Society for Heart and Lung Transplantation (ISHLT) consensus guidelines [5]. The molecular response was analyzed by plotting the temporal distribution of cytokine levels within the first 72 h after reperfusion in the 40-patient subgroup. Our secondary aim was to compare clinical outcomes between men and women after lung transplantation.

### Sample collection

To ensure maximal consistency, we followed a standardized protocol for peripheral blood collection, processing, and storage. Ten milliliters of peripheral blood were collected (in collection tubes containing ethylenediaminetetraacetic acid [EDTA]) before transplant, described as baseline or T0, and 6, 24, 48, and 72 h after lung reperfusion. The blood samples were immediately transferred to the Texas Heart Institute Biorepository for biomarker analysis.

### Immunologic analyses

For biomarker analysis, blood collection tubes were centrifuged, and the plasma was isolated and immediately flash frozen and stored at − 80 °C. After slowly thawing on ice, plasma samples were processed according to the manufacturer’s recommendations for multiplex bead array (Bio-Plex, Bio-Rad Laboratories, Hercules, CA, USA). The plates were read with the Luminex MAGPIX with a lower limit of 100 beads per sample per analyte, and the cytokine concentrations were analyzed with the Bio-plex Results Generator. A coefficient of variation < 20% was the criterion for acceptance.

### Statistical analysis

#### Clinical analysis

**Fig. 2:**
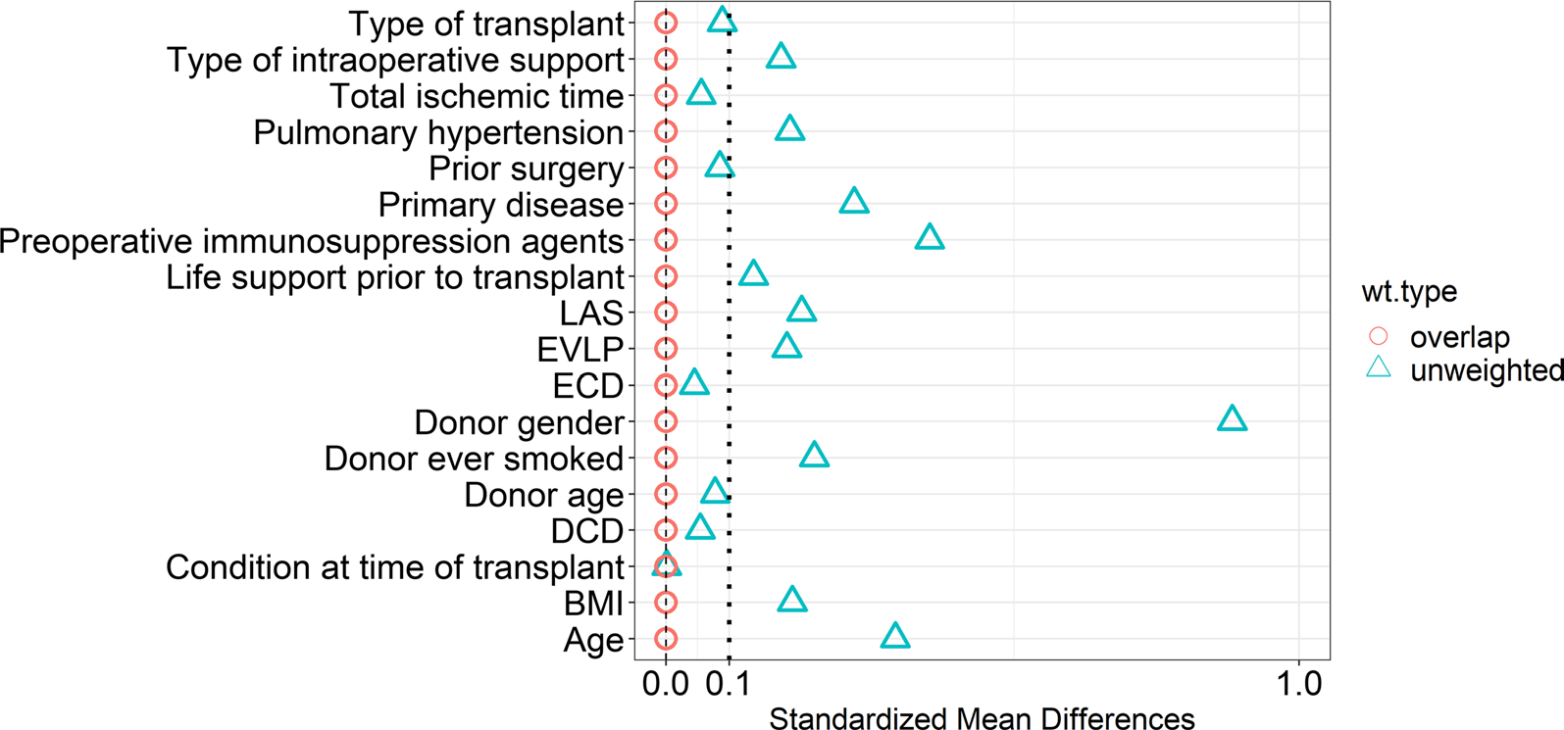
Standardized mean differences between unweighted data and overlap propensity weighted data. *BMI* body mass index, *DCD* donation after circulatory death, *ECD* extended criteria donor, *ELVP* ex vivo lung perfusion, *LAS* lung allocation score

All descriptive statistics are reported as percentages or means. Continuous variables with normal distribution were compared by using the two-sided Student t-test. Continuous variables with skewed distribution were compared by using the Wilcoxon rank-sum test. Categorical variables were compared by using the Chi-square test or Fisher exact test. Overlap propensity weighting analysis was used to adjust for factors that could affect the clinical outcomes (Fig. 2). Overlap propensity weighting achieves exact balance in the means of confounding variables by weighting each sample proportional to its propensity score [13, 14].

#### Cytokine analysis

We used analysis of variance (ANOVA) to compare longitudinal cytokine data. We fit a linear mixed-effects model (LMM) for each cytokine level and cell population. Because time and cytokine level are not linearly associated, a B-spline based on time, ${f}_{B}\left(time\right)$, was used to induce nonlinear structure. Moreover, ${f}_{B}\left(time\right)$, sex, and ${f}_{B}\left(time\right)sex$ were used as fixed effects, and random effects were allowed across subjects. ${f}_{B}\left(time\right)\times sex$ captures whether cytokine expression differs by the interaction between time and sex. To reduce residual errors, the LMMs were fitted on a log scale when there were no missing data. Overlap weighting was also used to adjust for three factors: type of transplant (single vs bilateral), ex vivo lung perfusion, and type of intraoperative ECLS [13, 14]. We used overlap weighting to achieve exact balance between groups in the means of confounding variables. ANOVA was used to test for differences between LMM coefficients of ${f}_{B}\left(time\right)\times sex$, and the ANOVA p-values were adjusted by using the Benjamini–Hochberg procedure for the original and overlap weighted cohorts, respectively. LMM coefficients ${f}_{B}\left(time\right)\times female-{f}_{B}\left(time\right)\times male$ that were far from zero would suggest that cytokine evolution is significantly different between female and male patients. Statistical analyses were conducted in R version 4.0.3 (R Foundation for Statistical Computing, Vienna, Austria). A two-sided p-value < 0.05 was considered significant.

## Results

### Clinical characteristics

The clinical cohort consisted of 203 patients: 122 men (60%) and 81 women (40%). There were several differences between men and women in terms of clinical, donor, and operative characteristics (Table 1). These differences were resolved after overlap propensity score weighting (Table 2). Of note, we did not notice a difference between men and women in the use of ECLS (75% vs 80%, p = 0.398) or EVLP (11% vs 14%, p = 0.250). We also did not notice any sex differences in intraoperative RBC transfusion, volume administration, or type of fluids administered.

**Table 1:** Demographic and clinical characteristics of 203 lung transplant recipients

	Overall (n = 203)	Male (n = 122)	Female (n = 81)	*p* value
Preoperative characteristics				
Age, y	54 ± 15	56 ± 14	51 ± 16	0.020
BMI, kg/m^2^	25 ± 5	26 ± 5	25 ± 6	0.170
Primary disease				
Cystic fibrosis or COPD	80 (39)	41 (66)	39 (52)	0.029
Restrictive lung disease	123 (61)	81 (34)	42 (48)	
Pulmonary vascular disease	7 (3)	2 (2)	5 (6)	
Lung allocation score	44 ± 14	45 ± 15	43 ± 10	0.766
Condition at time of transplant				
Hospitalized or in ICU	25 (12)	15 (12)	10 (12)	1.0
Not hospitalized	178 (88)	107 (88)	71 (88)	
Life support before transplant^a^	20 (10)	14 (12)	6 (7)	0.472
Pulmonary hypertension^b^	150 (74)	86 (71)	64 (79)	0.234
Mean PAP, mmHg	27 (10)	26 (10)	28 (9)	0.194
Preoperative immunosuppression agents	83 (41)	40 (33)	43 (53)	0.005
Type of immunosuppression				
Mycophenolate	12 (14)	4 (10)	8 (19)	0.611
Steroid	43 (52)	20 (50)	23 (53)	
Steroid + mycophenolate	19 (23)	10 (25)	9 (21)	
Steroid + mycophenolate + tacrolimus	4 (5)	3 (8)	1 (2)	
Others	5 (6)	3 (8)^c^	2 (5)^d^	
Prior surgery	47 (23)	30 (25)	17 (21)	0.612
Prior cardiac surgery	4 (2)	3 (2)	1 (1)	1.0
Prior lung surgery^e^	36 (18)	24 (20)	12 (15)	0.45
Prior lung transplant	3 (1)	2 (2)	1 (1)	1.0
Donor characteristics				
Age, y	35 ± 13	35 ± 13	36 ± 13	0.587
Extended criteria donor^f^	80 (39)	47 (39)	33 (41)	0.865
Ever smoked	107 (53)	70 (57)	37 (46)	0.136
Donor sex				
Male	127 (63)	96 (79)	31 (38)	< 0.001
Female	86 (37)	26 (21)	50 (62)	
Donation after circulatory death	11 (5)	6 (5)	5 (6)	0.710
Ex vivo lung perfusion	27 (13)	13 (11)	14 (17)	0.250
Chest radiography findings				
Not abnormal	120 (60)	73 (60)	47 (58)	0.870
Abnormal single lung	39 (19)	22 (18)	17 (21)	
Abnormal both lungs	44 (22)	27 (22)	17 (21)	
Respiratory secretions				
None or scant^g^	143 (72)	89 (74)	54 (69)	0.180
Thick but clear	52 (26)	28 (23)	24 (31)	
Repooling	3 (2)	3 (3)	0 (0)	
Bronchoscopy findings^h^				
Not abnormal	136 (69)	88 (73)	48 (62)	0.257
Abnormal^i^ single lung	22 (11)	11 (9)	11 (14)	
Abnormal both lungs	36 (18)	18 (15)	18 (23)	
Abnormal anatomy/other lesions	4 (2)	3 (3)	1 (1)	
Operative details				
Type of transplant				
Single	42 (21)	27 (22)	15 (19)	0.656
Bilateral	161 (79)	95 (78)	66 (82)	
Type of intraoperative support				
Off-pump	47 (23)	31 (25)	16 (20)	0.443
CPB	124 (61)	71 (58)	53 (65)	
Modified bypass	18 (9)	13 (11)	5 (6)	
ECMO	14 (7)	7 (6)	7 (9)	
Total ischemic time, min	339 ± 136	336 ± 124	343 ± 153	0.673
Units of RBC				
< 2	115 (57)	74 (61)	41 (51)	0.086
2–5	72 (35)	36 (30)	36 (44)	
6–10	12 (6)	10 (8)	2 (2)	
> 10	4 (2)	2 (2)	2 (2)	
Total intraoperative fluid volume, mL	3201 ± 1837	3372 ± 2038	2943 ± 1450	0.215
Type of intraoperative fluids^j^				
Colloids	31	19	12	0.713
Crystalloids	1	1	0	
Combination	170	102	68	

Data reported as n (%) or mean ± standard deviation

*BMI* body mass index, *COPD* chronic obstructive pulmonary disease, *CPB* cardiopulmonary bypass, *ECMO* extracorporeal membrane oxygenation, *ICU* intensive care unit, *PAP* pulmonary artery pressure, *RBC* red blood cells

^a^Life support before transplant was by ventilator, noninvasive positive-pressure ventilation, or ECMO

^b^Mean pulmonary artery pressure > 20 mmHg

^c^Others: steroid + mycophenolate + cyclophosphamide, steroid + methotrexate, or adalimumab

^d^Others: azathioprine or steroid + azathioprine

^e^Prior lung surgery does not include lung transplant

^f^Age > 55 y, anticipated ischemia > 6 h, donation after circulatory death, PaO_2_/FiO_2_ < 300, > 20 PYH smoker

^g^Data not available for 2 men and 3 women

^h^Data not available for 2 men and 3 women

^i^Purulent or blood secretion during bronchoscopy

^j^One woman did not receive any type of fluids intraoperatively

**Table 2:** Overlap weighted demographic and clinical characteristics of 203 lung transplant recipients

	Overall (n = 203)	Male (n = 122)	Female (n = 81)
Preoperative characteristics			
Age, y	54	54	54
BMI, kg/m^2^	25	25	25
Primary disease			
Cystic fibrosis or COPD	88 (44)	53 (44)	35 (44)
Restrictive lung disease or pulmonary vascular disease	115 (56)	69 (56)	46 (56)
Lung allocation score	44	44	44
Condition at time of transplant			
Hospitalized or in ICU	27 (13)	16 (13)	11 (13)
Not hospitalized	176 (87)	106 (87)	70 (87)
Life support before transplant^a^	18 (9)	11 (9)	7 (9.)
Pulmonary hypertension^b^	157 (77)	94 (77)	63 (77)
Preoperative immunosuppression agents	93 (46)	56 (46)	37 (46)
Prior surgery	48 (24)	29 (24)	19 (24)
Donor characteristics			
Age, y	35	35	35
Extended criteria donor^c^	85 (42)	51 (42)	34 (42)
Ever smoked	97 (48)	58 (48)	39 (48)
Donor sex			
Male	82 (40)	49 (40)	33 (40)
Female	122 (60)	73 (60)	49 (60)
Donation after circulatory death	13 (6)	8 (6)	5 (6)
Ex vivo lung perfusion	25 (13)	15 (13)	10 (13)
Operative details			
Type of transplant			
Single	162 (80)	97 (80)	65 (80)
Bilateral	41 (20)	25 (20)	16 (20)
Type of intraoperative support^d^	0.9	0.9	0.9
Total ischemic time, min	330	330	330

Data reported as n (%) or overlap weighted mean

*BMI* body mass index, *COPD* chronic obstructive pulmonary disease, *CPB* cardiopulmonary bypass, *ECMO* extracorporeal membrane oxygenation, *ICU* intensive care unit

^a^Life support before transplant was by ventilator, noninvasive positive-pressure ventilation, or ECMO

^b^Mean pulmonary artery pressure > 20 mmHg

^c^Age > 55 y, anticipated ischemia > 6 h, donation after circulatory death, PaO_2_/FiO_2_ < 300, > 20 PYH smoker

^d^Overlap weighed mean with 0 = CPB, 1 = ECMO, 2 = modified bypass, 3 = off-pump

### Clinical response to ischemia reperfusion

#### Grade 3 PGD at 48 and 72 h

In the full cohort, grade 3 PGD (PGD3) at T48 was more common in men (n = 39; 32%) than in women (n = 15; 18.5%). After overlap propensity score weighting for both men and women, the PGD3 rate at T48 remained higher in men (35.6% vs 20.7%). The overlap propensity score–weighted average treatment effect among the overlap population (ATO) was − 0.149 (95% CI: − 0.274, − 0.024), suggesting that fewer women than men had PGD3 at T48 (Tables 3, 4).

**Table 3:** Outcomes of 203 lung transplant recipients

	Overall (n = 203)	Male (n = 122)	Female (n = 81)	*p* value
Primary outcomes				
PGD3 at T48	54 (27)	39 (32)	15 (19)	0.050
PGD3 at T72	47 (23)	30 (25)	17 (21)	0.670
PGD decrease from T0 to T72	61 (30)	30 (25)	31 (38)	0.087
PGD difference from T72 to T0	− 0.084 ± 0.95	0.016 ± 0.94	− 0.235 ± 0.95	0.047
Secondary outcomes				
Postoperative bacteremia	55 (27)	25 (21)	30 (37)	0.015
Postoperative wound infection	6 (3)	3 (3)	3 (4)	0.685
* C. difficile* colitis	5 (3)	2 (2)	3 (4)	0.390
90-d survival	197 (97)	117 (96)	80 (99)	0.405
1-y survival	142 (89), n = 159	87 (88), n = 99	55 (92), n = 60	0.628
Peak lactate within 72 h, mg/dL	7 ± 4, n = 188	7 ± 4, n = 113	6 ± 3, n = 75	0.002
Stroke	6 (3)	4 (3)	2 (3)	1
Acute rejection during hospitalization	18 (9)	12 (10)	6 (7)	0.731
ICU LOS, d	16 ± 23	16 ± 24	17 ± 25	0.966
Tracheostomy	44 (22)	24 (20)	20 (25)	0.499
Airway dehiscence	5 (3)	3 (3)	2 (3)	1
Mechanical ventilation ≥ 5 d	54 (27)	29 (24)	25 (31)	0.338
Postoperative ECMO	22 (11)	15 (12)	7 (9)	0.556
Readmission within 1 y	158 (89), n = 177	99 (90), n = 109	59 (87), n = 68	0.549

Data reported as n (%) or mean (standard deviation)

*ECMO* extracorporeal membrane oxygenation, *ICU* intensive care unit, *LOS* length of stay, *PGD* primary graft dysfunction

**Table 4:** Association between sex and primary and secondary outcomes: Overlap propensity score-weighted average treatment effect among the overlap population (ATO)

	ATO (95% CI)	*p* value
Primary outcomes		
PGD3 at T48	− 0.143 (− 0.290, − 0.028)	0.055
PGD3 at T72	− 0.065 (− 0.204, 0.075)	0.365
PGD decrease from T0 to T72	0.193 (0.048, 0.337)	0.009
PGD difference from T72 to T0	− 0.319 (− 0.631, − 0.007)	0.045
Secondary outcomes		
Postoperative bacteremia	0.072 (− 0.063, 0.207)	0.295
Postoperative wound infection	0.020 (− 0.054, 0.094)	0.601
* C. difficile* colitis	0.055 (− 0.014, 0.124)	0.119
90-d survival	0.042 (− 0.017, 0.100)	0.164
1-y survival	0.040 (− 0.093, 0.172)	0.555
Peak lactate within 72 h, mg/dL	− 1.565 (− 2.622, − 0.509)	0.004
Stroke	0.007 (− 0.066, 0.080)	0.854
Acute rejection during hospitalization	− 0.052 (− 0.162, 0.059)	0.362
ICU LOS, d	3.249 (− 4.766, 11.263)	0.427
Tracheostomy	0.126 (− 0.013, 0.265)	0.075
Airway dehiscence	− 0.012 (− 0.072, 0.049)	0.703
Mechanical ventilation ≥ 5 d	0.130 (− 0.010, 0.270)	0.069
Postoperative ECMO	− 0.038 (− 0.149, 0.074)	0.507
Readmission within 1 y	− 0.029 (− 0.140, 0.082)	0.607

Data reported as ATO (95% CI). 95% CI not including 0 indicates a statistically significant difference in secondary outcome between men and women

*ECMO* extracorporeal membrane oxygenation, *ICU* intensive care unit, *LOS* length of stay, *PGD* primary graft dysfunction

In the full cohort, the rate of PGD3 at T72 was similar between men (n = 30; 24.6%) and women (n = 17; 21.0%). After overlap propensity score weighting, the PGD3 rate at T72 was 29.3% in men and 22.8% in women. The ATO was − 0.065 (95% CI: − 0.204, 0.075), suggesting that there was no association between sex and PGD3 at T72.

Of note, the rate of combined PGD2 and PGD3 at T48-72 h was 70% (85/122) for men and 73% (59/81) for women (p = 0.640).

#### Grade 3 PGD from 0 through 72 h

**Fig. 3:**
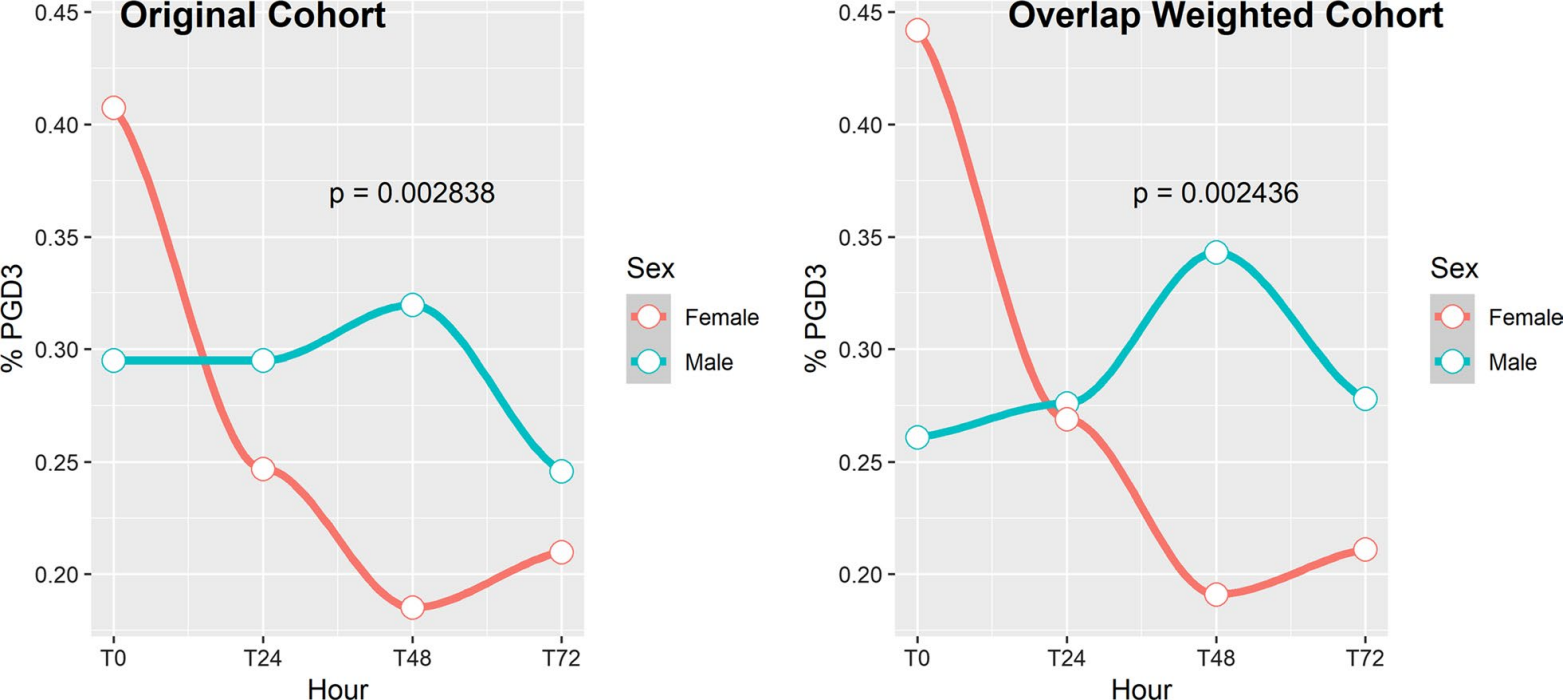
Evolution of PGD within 72 h after reperfusion in men (green) and women (red). p values are unadjusted

Figure 3 shows the distribution of PGD3 events at 0, 24, 48 and 72 h after reperfusion in men and women. Reductions in PGD3 over time were less common in male patients (n = 30; 24.6%) than in female patients (n = 31; 38.3%). This difference was even greater after overlap propensity score weighting: 19.7% in men versus 38.9% in women. The ATO was 0.193 (95% CI: 0.048, 0.337), suggesting that PGD more often decreased in women than in men.

Next, we examined whether the evolution of PGD, defined as PGD score (on a scale of 0–3) at T72 minus PGD score at T0, was different between sexes. The mean PGD difference from T72 to T0 was 0.016 for men and − 0.235 for women. After overlap propensity score weighting for both men and women, the mean PGD difference from T72 to T0 was 0.133 for men and − 0.186 for women. The overlap propensity score–weighted ATO was − 0.319 (95% CI: − 0.631, − 0.007), which supports the hypothesis that the evolution of PGD was different between men and women, favoring a better outcome in women. Additionally, we explored the average number of living children and PGD score 3 at each timepoint in the female cohort. However, no differences were found at T0 (1.52 ± 1.31 vs 1.91 ± 1.85, p = 0.476), T24 (2.05 ± 1.36 vs 1.64 ± 1.73, p = 0.17), T48 (1.87 ± 1.20 vs 1.72 ± 1.75, p = 0.46) and T72 (2.06 ± 1.59 vs 1.66 ± 1.60, p = 0.30).

### Cytokine response to ischemia reperfusion

A subgroup of 40 patients had peripheral blood samples available for cytokine analysis. The 27 male and 13 female patients in this subgroup had no statistically significant differences between them in clinical characteristics except for greater percentages of restrictive lung disease and preoperative use of anti-inflammatory agents among the male patients (Additional file 1: Table S1).

**Fig. 4:**
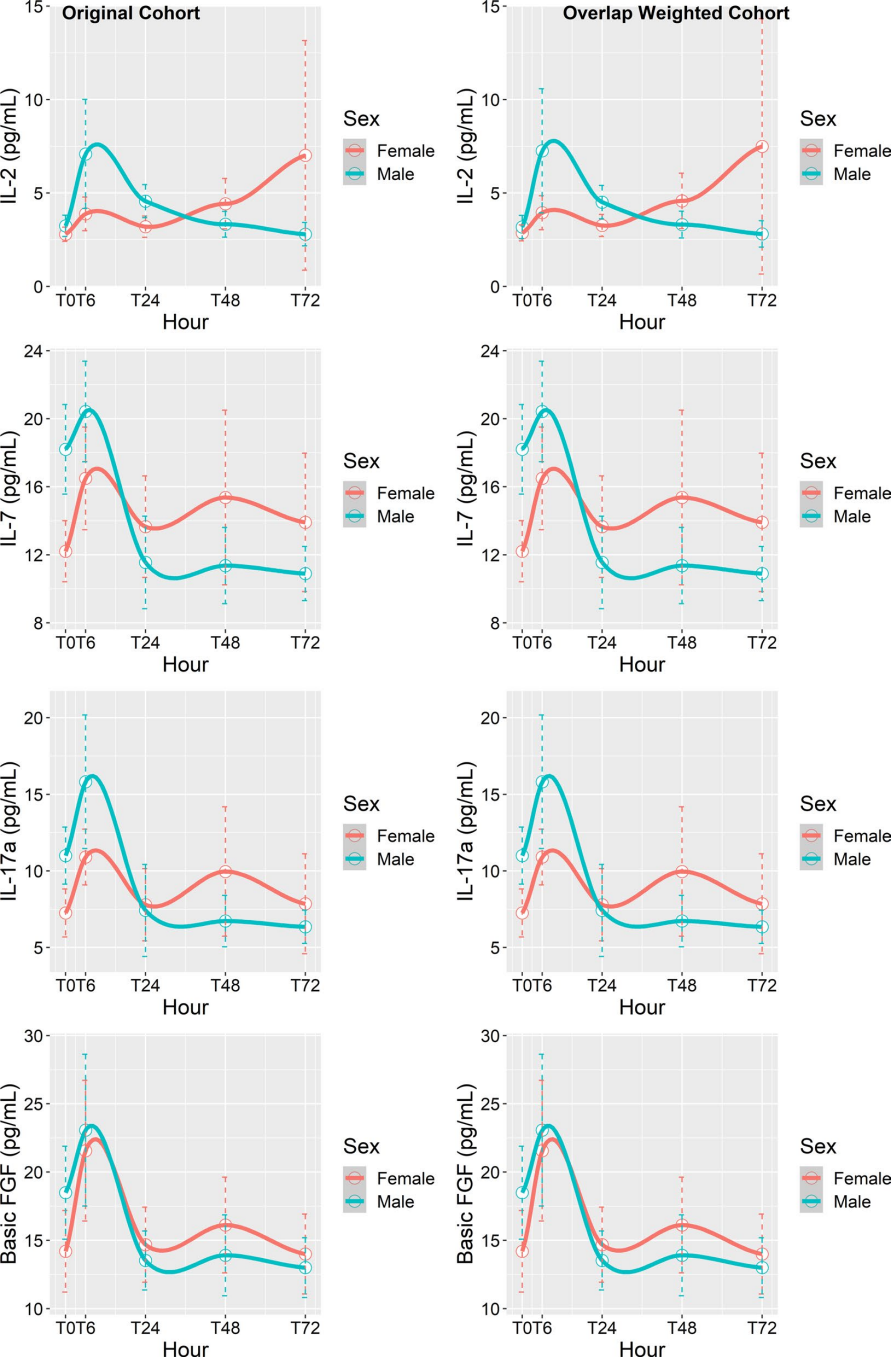
Evolution of several cytokines in the 40-patient subgroup that were different between men (green) and women (red)

We found that sex had a statistically significant effect on the evolution of IL-2, IL-7, IL-17a, and basic fibroblast growth factor (B-FGF) (Fig. 4). This effect remained significant after adjustment for multiple comparisons in both the original and overlap weighted cohorts (Additional file 2: Table S2). Reviewing the adjusted p-values suggested that sex did not have a significant effect on the evolution of the following cytokines: TNF-α, IL-1B, IL-1ra, MCP-1, RANTES, MIP-1A, MIP-1B, IL-9, PDGF-BB, IP-10, eotaxin, IL-4, and G-CSF (Additional file 2: Table S2).

### Secondary clinical outcomes

We compared outcomes in the full cohort (n = 203) between men and women (Table 3). Overlap propensity score weighting was used to determine whether these differences were significant after adjustment for potentially confounding variables (Table 4). The mean peak lactate within 72 h was 7.39 mg/dL for men and 5.61 mg/dL for women. After overlap propensity score weighting, the mean peak lactate within 72 h was 6.99 mg/dL in men and 5.43 mg/dL in women. The overlap score-weighted average treatment effect among the overlap population (ATO) was − 1.57 (95% CI: − 2.622, − 0.509) suggesting that women had lower mean peak lactate elevations within 72 h after lung transplantation than men.

We did not observe statistically significant differences between men and women with regard to postoperative bacteremia, wound infections, *Clostridium difficile* colitis, 90-day survival, 1-year survival, stroke rate, acute rejection, postoperative intensive care unit LOS, tracheostomy use, airway dehiscence, prolonged ventilation, postoperative ECMO use, or rehospitalizations (Tables 3, 4).

## Discussion

The current study is the first, to our knowledge, to explore sex-based differences in the evolution of clinical and molecular features of IR after lung transplantation. We noted more frequent resolution of PGD, the clinical phenotype of IR, in women than in men, even though women had a more robust and sustained expression of proinflammatory cytokines (IL-2, IL-7, IL-17a, and B-FGF). These findings show sex-specific differences in lung IR, which raises the potential for tailoring management to improve lung transplant outcomes in both sexes.

Our data suggest that women had less severe PGD than men. Women had less PGD3 at 48 h. Although there was no significant sex difference in PGD3 at 72 h, PGD3 at 72 h is less common in both sexes, making it more difficult to detect differences. Compared with men, between 0 and 72 h, women had a greater reduction in the incidence of PGD3 and greater improvement in their mean PGD score (0–3).

Long-term outcomes after lung transplantation can be significantly affected by even transient PGD [15]. If women do, in fact, have an inherent protective mechanism against severe IR injury and PGD, this could explain the superior long-term outcomes reported in several clinical series [1–3].

These findings have other important implications related to recipient and donor selection. Female patients on average have shorter stature than men, making it more difficult to find a donor lung of appropriate size; this could lead to longer wait times and, therefore, poorer survival on the transplant waitlist. This is especially true in patients with restrictive lung disease [16–18]. Another factor leading to longer wait times is related to underutilization of donor organs. Donor factors such as smoking history and other extended criteria features could increase recipients’ risk of PGD [4, 19, 20]. It is conceivable that female recipients are less susceptible to the deleterious effects of these donor risk factors, which would allow a broader consideration of donor organs for women patients.

Our subgroup cytokine analysis showed sex-specific differences in molecular responses to IR after lung transplantation. IL-2 levels rose during the first 6 h in both men and women, but whereas men had a greater elevation at 6 h with a return to baseline by 24–48 h, women had a return to baseline at 24 h, followed by a gradual rise for up to 72 h (Fig. 3). IL-2 is an X-linked cytokine produced by T-cells and antigen-presenting cells and is involved in stimulating T-cell proliferation and activation. This could have two different implications. One is that T-cell activation is implicated in the pathogenesis of PGD and could explain how the earlier and more robust rise in IL-2 at 6 h in men results in more severe PGD than women have [8]. On the other hand, IL-2 has also been shown to stimulate regulatory T-cell activity, which may be protective against PGD and rejection [21, 22]. This could explain why a gradual and sustained elevation in IL-2 coincides with resolution of PGD in women.

In addition to IL-2, three other cytokines showed a different evolution after reperfusion in women versus men. IL-7 had a similar expression pattern to IL-2’s after reperfusion: a marked resolution in men, contrasting with a persistent rise in women. While IL-7 has several distinct features, it may protect against PGD through T-cell–mediated events similar to those associated with IL-2 [23]. IL-17 had a similar distribution after reperfusion and also has both protective and destructive T-cell–mediated inflammatory effects [24]. B-FGF expression followed a similar trend to that of IL-7, with the exception that its peak at 6 h was similar between the sexes. B-FGF levels remained more elevated in women than men at 48–72 h. B-FGF is a growth factor produced by a wide variety of cells but one that has proangiogenic effects in the lung. It is conceivable that elevations in B-FGF after IR could improve endothelial cell function and integrity, which is vital for recovery from PGD. Conversely, overproduction of B-FGF has been shown to result in pulmonary hypertension and fibrosis [25].

Sex-based differences in IR have been noted by others in patients with cardiac, renal, and liver injury [26–30]. Our study showed sex-based differences in the clinical phenotype of IR injury (PGD), as well as IR-induced cytokine production, after lung transplantation. Moreover, we noticed a sex-based difference in lactate production after reperfusion. Lactate is produced by several end organs in response to IR [31–34]. Our observation that peak lactate levels were lower in women than men further supports the theory that women are better protected against IR.

This protection from IR may not always translate into meaningful clinical differences. For instance, we did not see a difference between men and women in terms of postoperative bacteremia, wound infections, *Clostridium difficile* colitis, 90-day survival, 1-year survival, stroke, acute rejection, postoperative intensive care unit LOS, tracheostomy use, airway dehiscence, prolonged ventilation, postoperative ECMO use, or rehospitalizations. It is possible that the lack of significant differences in clinical outcomes was due to sample size. But previous analyses of sex-based outcomes in the era before the lung allocation score, when transplants were not prioritized by recipient urgency, also found no clinically significant difference in long-term survival between men and women [3, 35]. Thus, while our study showed sex-based differences in susceptibility to IR injury in favor of women, the extent to which these differences are clinically relevant may depend on a variety of additional perioperative factors.

### Limitations

Clinical observational studies are subject to potential, unrecognized confounders that could bias results. PGD grading can be subject to bias and interrater variability. Because of this, we used a rigorous method of determining PGD by using an expert grader and strictly adhering to the ISHLT 2016 guidelines for intubated and extubated patients. While we realize that PGD2 is also an important outcome, our analysis of the temporal evolution of PGD was limited to detecting extreme changes such as PGD3 to PGD0 or PGD1. Thus, we were not able to thoroughly explore differences in PGD2 between men and women. Also, T0 PGD scores accounted for the main difference between male and female patients, and without this measurement, it is conceivable that the differences between men and women would be marginal. This time point is controversial but still supported by the 2016 ISHLT consensus on PGD scoring. In addition, the peak cytokine expression occurred at this time point, making it an important time point for this study. The distribution of potential confounders for PGD scoring at T0, such as use of ECLS and EVLP, was similar between groups. Additionally, we acknowledge the importance of the hormonal status of the female patients; unfortunately, these data were not available.

Furthermore, the subgroup of 40 patients with blood samples available had a greater prevalence of restrictive lung disease and preoperative use of anti-inflammatory agents than the full cohort. Whether the patient’s primary disease alone could affect the evolution of cytokine levels over time requires further study. It is possible to make a type II error by incorrectly concluding that a particular cytokine did not show a difference in evolution between men and women. For instance, G-CSF, IL-4, IP-10, and eotaxin all differed significantly between men and women in their temporal evolution, but this significance was lost after adjustment for multiple comparisons (Additional file 2: Table S2). Similarly, limited sample size could have hampered our efforts to find meaningful differences in clinical outcomes. Further studies with larger numbers of patients could help clarify these effects. Finally, this analysis sought to show differences in molecular markers of IR through cytokine expression; we did not intend to, nor did we, establish a causal correlation between cytokine expression and PGD.

## Conclusions

In the current study, we found several notable differences between male and female patients in the evolution of PGD and cytokine production after lung transplantation. Women showed a more sustained proinflammatory response than men despite a greater reduction in PGD over time. The relationship between these sex-based differences in cytokine production and PGD requires additional investigation. Our study adds to the growing body of evidence for sex-based differences in disease pathophysiology and clinical outcomes. Whether sex-specific therapies or treatment protocols in lung transplantation could improve outcomes warrants further evaluation.

## Supplementary Information

**Additional file 1:** Demographic and clinical characteristics of 40 lung transplant recipients with prospectively collected biomarkers.

**Additional file 2:** P values for testing differences between coefficients of *f*_*B*_ (*time*) × *sex*.

## Data Availability

The datasets used and analyzed in the current study are available from the corresponding author on reasonable request.

## Abbreviations

ANOVA: Analysis of variance
ECMO: Extracorporeal membrane oxygenation
EDTA: Ethylenediaminetetraacetic acid
B-FGF: Basic fibroblast growth factor
IR: Ischemia–reperfusion
ISHLT: International Society for Heart and Lung Transplantation
