# Correction to: Analysis of sex-based differences in clinical and molecular responses to ischemia reperfusion after lung transplantation

**DOI:** 10.1186/s12931-022-02152-0

**Published:** 2022-09-07

**Authors:** Lourdes Chacon-Alberty, Shengbin Ye, Daoud Daoud, William C. Frankel, Hassan Virk, Jonathan Mase, Camila Hochman-Mendez, Meng Li, Luiz C. Sampaio, Doris A. Taylor, Gabriel Loor

**Affiliations:** 1 Department of Regenerative Medicine, Texas Heart Institute, Houston, TX USA; 2 Department of Statistics, Rice University, Houston, TX USA; 3 Division of Cardiothoracic Transplantation and Circulatory Support, Michael E. DeBakey Department of Surgery, Baylor College of Medicine, Houston, TX USA; 4 Department of Cardiopulmonary Transplantation and Center for Cardiac Support, Texas Heart Institute, 6770 Bertner Ave, Suite 355-K, Houston, TX 77030 USA; 5 Present Address: Division of Infectious Diseases, Department of Internal Medicine, Center for Antimicrobial Resistance and Microbial Genomics (CARMiG), University of Texas Health Science Center at Houston, Houston, TX USA; 6 Present Address: Department of Advanced Cardiopulmonary Therapies and Transplantation, University of Texas Health Science Center at Houston, Houston, TX USA; 7 Present Address: RegenMedix Consulting, Houston, TX USA

## Correction to: Respiratory Research (2021) 22:318 10.1186/s12931-021-01900-y

Following publication of the original article [1], the authors identified an error in affiliation and grant number is missing in the funding.

**Funding**

This research is supported by grants from the Roderick D. MacDonald Research Fund (Grant Number 20RDM004), the JLH Foundation (Grant Number 55354), and the NIH T32 fund (Grant Number T32CA096520).

The affiliation for Drs. **Meng Li** and **Shengbin Ye** should be Department of Statistics, not Department of Biostatistics.
