# Supplementary material for: Analysis of sex-based differences in clinical and molecular responses to ischemia reperfusion after lung transplantation
Source: Respir Res. 2021 Dec 22;22:318. doi: 10.1186/s12931-021-01900-y (PMC8693497; doi:10.1186/s12931-021-01900-y)
Supplement: Supplementary file 1 — Additional file 1: Demographic and clinical characteristics of 40 lung transplant recipients with prospectively collected biomarkers. [file 12931_2021_1900_MOESM1_ESM.docx]

**Supplementary Table 1** Demographic and clinical characteristics of 40 lung transplant recipients with prospectively collected biomarkers

|  | Overall  (n=40) | Male  (n=27) | Female  (n=13) | *P* value |
| --- | --- | --- | --- | --- |
| Preoperative characteristics | | | | |
| Age, y | 52 ± 15 | 54 ± 14 | 47 ± 16 | .319 |
| BMI, kg/m^2^ | 26 ± 5 | 27 ± 5 | 24 ± 5 | .081 |
| Primary disease |  |  |  | .017 |
| Cystic fibrosis or COPD | 19 (48) | 9 (33) | 10 (77) |  |
| Restrictive lung disease or pulmonary vascular disease | 21 (53) | 18 (67) | 3 (23) |  |
| Lung allocation score | 43 ± 12 | 44 ± 14 | 40 ± 6 | .189 |
| Condition at time of transplant |  |  |  | 1 |
| Not hospitalized | 37 (93) | 25 (93) | 12 (92) |  |
| In ICU | 3 (8) | 2 (7) | 1 (8) |  |
| Life support before transplant^a^ | 4 (10) | 2 (7) | 2 (15) | .584 |
| Pulmonary hypertension^b^ | 29 (73) | 20 (74) | 9 (69) | 1 |
| Preoperative immunosuppression agents | 10 (25) | 6 (22) | 4 (31) | .700 |
| Prior surgery | 6 (15) | 5 (19) | 1 (8) | .643 |
| Donor characteristics |  |  |  |  |
| Age, y | 39 ± 13 | 38 ± 14 | 39 ± 13 | .751 |
| Extended criteria donor^c^ | 19 (48) | 11 (41) | 8 (62) | .314 |
| Ever smoked | 22 (55) | 17 (63) | 5 (39) | .185 |
| Donor sex |  |  |  | <.001 |
| Male | 28 (70) | 24 (89) | 4 (31) |  |
| Female | 12 (30) | 3 (11) | 9 (69) |  |
| Ex vivo lung perfusion | 11 (28) | 7 (26) | 4 (31) | 1 |
| Operative details | | | | |
| Type of transplant |  |  |  | .690 |
| Single | 9 (23) | 7 (26) | 2 (15) |  |
| Bilateral | 31 (78) | 20 (74) | 11 (85) |  |
| Type of intraoperative support |  |  |  | .703 |
| Off-pump | 15 (38) | 11 (41) | 4 (31) |  |
| CPB | 12 (30) | 7 (26) | 5 (39) |  |
| Modified bypass | 13 (33) | 9 (33) | 4 (31) |  |
| Total ischemic time, min | 410 ± 168 | 411 ± 170 | 407 ± 170 | .773 |
| *BMI* body mass index, *COPD* chronic obstructive pulmonary disease, *CPB* cardiopulmonary bypass, *ECMO* extracorporeal membrane oxygenation, *ICU* intensive care unit  Data reported as n (%) or mean (standard deviation)  ^a^Life support before transplant was by ventilator, noninvasive positive-pressure ventilation, or ECMO  ^b^Mean pulmonary artery pressure > 20 mmHg  ^c^Age >55 y, anticipated ischemia >6 h, donation after circulatory death, PaO_2_/FiO_2_ <300, >20 PYH smoker | | | | |
