# Supplementary material for: Analysis of sex-based differences in clinical and molecular responses to ischemia reperfusion after lung transplantation
Source: Respir Res. 2021 Dec 22;22:318. doi: 10.1186/s12931-021-01900-y (PMC8693497; doi:10.1186/s12931-021-01900-y)
Supplement: Supplementary file 2 — Additional file 2: P values for testing differences between coefficients of fB (time) × sex. [file 12931_2021_1900_MOESM2_ESM.docx]

**Supplementary Table 2** P values for testing differences between coefficients of $f_{B}\left( time \right)\times sex$

| Cytokine population | Full cohort (n=40) | Overlap weighted cohort (n=40) |
| --- | --- | --- |
| Basic FGF | .009 (.042) | .009 (.040) |
| Eotaxin | .049 (.105) | .050 (.112) |
| G-CSF | .047 (.105) | .048 (.105) |
| IL-1$\boldsymbol{\beta}$ | .497 (.528) | .504 (.567) |
| IL-1ra | .404 (.458) | .397 (.477) |
| IL-2 | .008 (.042) | .006 (.042) |
| IL-4 | .022 (.072) | .021 (.076) |
| IL-7 | .001 (.014) | .001 (.015) |
| IL-9 | .747 (.747) | .748 (.748) |
| IL-17$\boldsymbol{\alpha}$ | .010 (.042) | .009 (.040) |
| IP-10 | .035 (.098) | .043 (.112) |
| MCP-1 | .119 (.202) | .118 (.213) |
| MIP-1$\boldsymbol{\alpha}$ | .057 (.109) | .057 (.114) |
| MIP-1$\boldsymbol{\beta}$ | .157 (.238) | .157 (.252) |
| PDGF-BB | .168 (.238) | .168 (.238) |
| RANTES | .215 (.262) | .217 (.279) |
| TNF-$\boldsymbol{\alpha}$ | .203 (.262) | .196 (.271) |
| Data reported as *P* value (BH-adjusted *P* value)  *P* value < 0.05 suggests statistically significant differences in cytokine evolution between male and female patients | | |
